# Revisiting de Beer’s textbook example of heterochrony and jaw elongation in fish: *calmodulin* expression reflects heterochronic growth, and underlies morphological innovation in the jaws of belonoid fishes

**DOI:** 10.1186/2041-9139-5-8

**Published:** 2014-02-05

**Authors:** Helen M Gunter, Claudia Koppermann, Axel Meyer

**Affiliations:** 1 Department of Biology, Lehrstuhl für Zoologie und Evolutionsbiologie, University of Konstanz, Universitätstrasse 10, 78457 Constance, Germany; 2 Zukunftskolleg, University of Konstanz, Universitätstrasse 10, 78457 Constance, Germany

**Keywords:** Heterochrony, Morphological innovation, Jaw development, Beloniformes, Gavin de Beer, *Dermogenys*, *Belone*, Medaka, *Calmodulin*

## Abstract

**Background:**

Heterochronic shifts during ontogeny can result in adaptively important innovations and might be initiated by simple developmental switches. Understanding the nature of these developmental events can provide insights into fundamental molecular mechanisms of evolutionary change. Fishes from the Suborder Belonoidei display a vast array of extreme craniofacial morphologies that appear to have arisen through a series of heterochronic shifts. We performed a molecular heterochrony study, comparing postembryonic jaw development in representatives of the Suborder Belonoidei, the halfbeak *Dermogenys pusilla* (where the lower jaw is considerably elongated compared to the upper jaw) and the needlefish *Belone belone* (where both jaws are elongated), to a representative of their sister group the Suborder Adrianichthyoidei, the medaka *Oryzias latipes*, which has retained the ancestral morphology.

**Results:**

Early in development, the lower jaw displays accelerated growth both in needlefish and halfbeak compared to medaka, and secondary acceleration of the upper jaw is seen in needlefish later in their development, representing a case of mosaic heterochrony. We identified toothless extensions of the dentaries as innovations of Belonoid fishes and the source of heterochronic growth. The molecular basis of growth heterochronies in the Belonoidei was examined through comparing expression of skeletogenic genes during development of halfbeak and medaka. The *calmodulin* paralogue *calm1* was identified as a potential regulator of jaw length in halfbeak as its expression gradually increases in the lower jaw, but not the upper jaw, in a pattern that matches its outgrowth. Moreover, medaka displays equal expression of *calm1* in the upper and lower jaws, consistent with the lack of jaw outgrowth in this species.

**Conclusions:**

Heterochronic shifts in jaw growth have occurred repeatedly during the evolution of Belonoid fishes and we identify toothless extensions of the dentaries as an important innovation of this group. Our results suggest that *calm1* contributes to jaw heterochrony in halfbeak, potentially driving further heterochronic shifts in jaw growth across the Suborder Belonoidei, such as the upper jaw acceleration observed in needlefish.

## Background

One of the major goals of evolutionary biology is to understand the developmental and genetic origins of morphological novelties that are of adaptive relevance [1]. Already classic work by Gavin De Beer advanced the idea that some novelties arise as the result of heterochronic shifts, which involve an alteration in the timing or rate of ontogenetic events during evolution, rather than having a completely *de novo* origin [2-8]. Although categorizations of heterochronic shifts have been devised (Additional file 1: Figure S1), they essentially involve - compared to the ancestral condition - either an increased growth rate or the addition of ontogenetic steps (peramorphosis), or the retardation of growth or a reduced number of ontogenetic steps (paedomorphosis) [9]. While heterochronic shifts in growth can result in dramatic alterations to adult morphology, due to the modular nature of development, such changes can be induced by simple developmental switches [10].

Increasingly, molecular developmental approaches are becoming incorporated into studies of heterochrony since they can shed light on the genetic bases of the heterochronic shifts and the regulatory changes that may underlie them [4,7,8,11]. By applying a comparative framework, molecular studies of heterochrony have the potential to identify not only the ontogenetic stage, structure and magnitude of growth of morphological alterations, but also their genetic bases. This approach involves developmental comparisons between members of different lineages to their sister lineages that retained the ancestral condition (for examples see [8,12-15]). Heterochronic growth may be underlain by various developmental mechanisms that alter the proliferation, differentiation or apoptosis of various cell populations and as such, identifying its genetic basis can be aided by a fine-scaled developmental approach [4,16]. Such developmental investigations have been successfully utilized in studies in cichlid fish and Galapagos finches, which interestingly, point at similar molecular drivers of differential proliferation in cellular condensations that contribute to shape differences in adult jaws and beaks respectively (that is, *bmp4* and *calmodulin*) [17-22].

For more than 100 years, due to their striking jaw morphologies beloniform fishes have been subjected to ontogenetic studies and, even more importantly, inspired giants in the field such as Severtzov, De Beer, and Gould to devise the theory of heterochrony ( [2,23]; [24-26] cited in [27]). In fact the beloniform example played an important role in the incorporation of Darwinian thinking into developmental biology and the founding of the field of evolutionary morphology (reviewed in [3,28]). In Severtzov’s classic treatment [29] he sorted out some of the confusions and misinterpretations of von Baer’s law and from the misguided interpretation of Haeckel.

**Figure 1:**
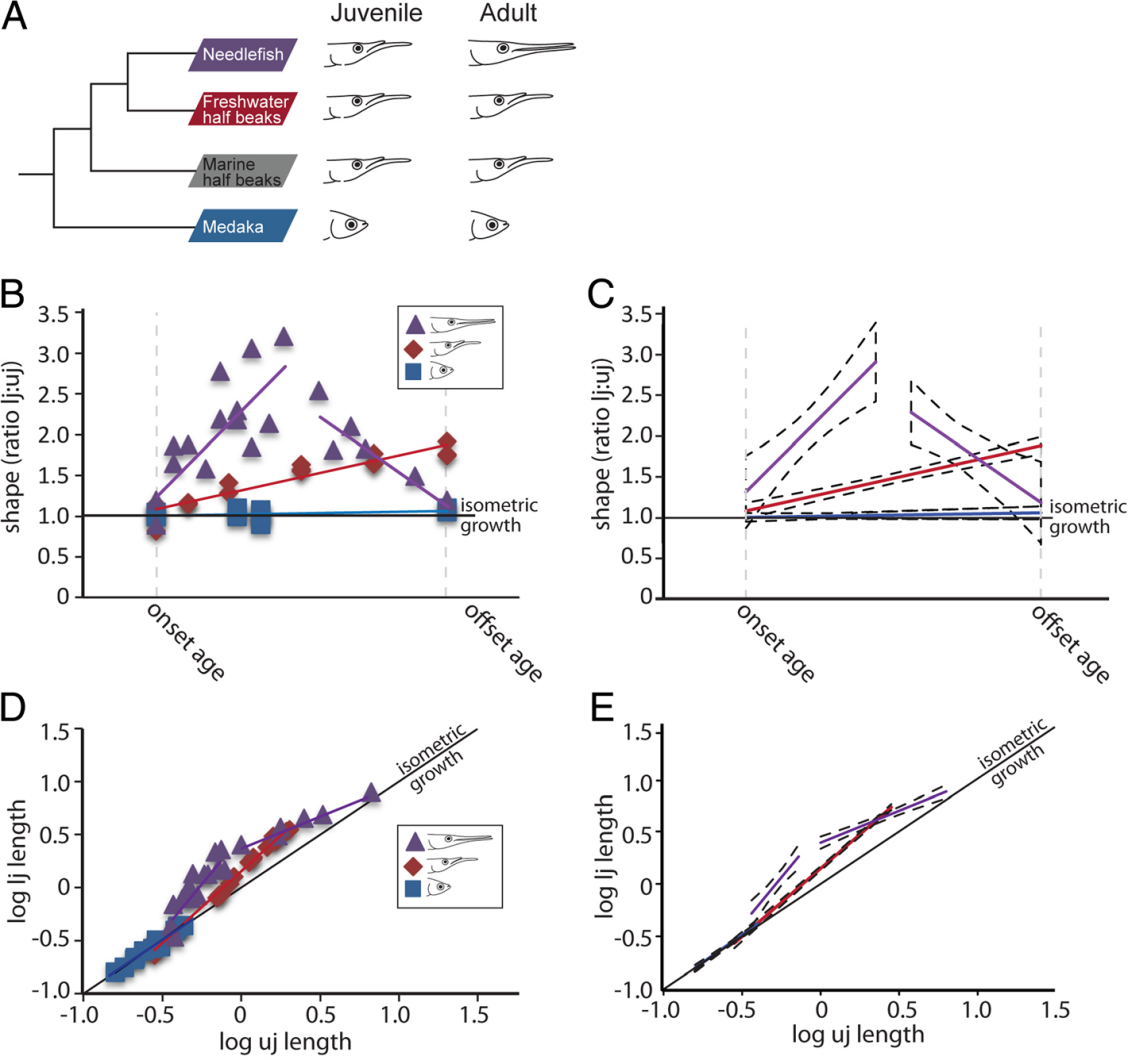
**Heterochrony has contributed significantly to craniofacial diversity in the Belonoidei. (A)** Simplified phylogeny based on [27] showing the relationships between representative taxa of the Belonoidei including needlefish and halfbeak, and medaka, which belong to their sister group the Adrianichthyoidei. **(B, C)** Developmental heterochrony was assessed in the jaws of these three species by plotting shape, represented by the ratio of lower jaw (lj) to upper jaw (uj) length (lj:uj), against relative age. The term ‘onset age’ refers to the commencement of skeletal ossification, while ‘offset age’ refers to the completion of ossification. Medaka retained the ancestral condition, maintaining an lj:uj of 1.0. Conversely, needlefish and halfbeak display acceleration of lj growth relative to the uj (indicated by lj:uj > 1.0). This ratio is maintained to adulthood in halfbeak. Additionally, needlefish display a secondary acceleration of uj growth, resulting in a ratio of approximately 1.0, which is then maintained until adulthood. **(D, E)** In addition to the aforementioned heterochronic shifts, the uj and lj also display allometry in their growth, as indicated by plotting their ontogenetic trajectories. **(C, E)** 95% confidence intervals are indicated for both the heterochrony plot and ontogenetic trajectory respectively.

The Order Beloniformes includes the Suborder Belonoidei, which contains such diverse representatives as the needlefishes (Belonidae), halfbeaks (Hemiramphidae), flying fishes (Exocoetidae) and sauries (Scomberosocidae). The single other Suborder in the Order Beloniformes is the Adrianichthyoidei, the ricefishes, which includes medaka, a developmental model system. The families in the Suborder Belonoidei differ remarkably in the relative lengths of their upper and lower jaws. Halfbeaks, as the name suggests, have elongated lower jaws and relatively short, non-protrusible upper jaws whereas needlefish have elongated upper and lower jaws of equal length. Interestingly, most needlefish pass through a ‘halfbeak’ stage as juveniles, which led to the hypothesis that halfbeaks might be paedomorphic derivatives of the needlefish ( [26] cited in [2,27]). This hypothesis was called into question however, by a molecular phylogeny of the Beloniformes, which formed the basis for a reconstruction of ontogenetic transitions in jaw length (Figure 1A) [27,30]. The secondary growth of the upper jaw of the needlefish would therefore best be interpreted as peramorphosis (specifically, hypermorphosis; see Additional file 1: Figure S1) rather than paedomorphosis.

Within the Belonoidei the ‘halfbeak’ morphology appears to represent the ancestral state, from which all other jaw morphologies are derived (including the secondary resorption of an initially elongated lower jaw in flying fish, as would be implied by the phylogeny). Interestingly, the members of the Suborder Adrianichthyoidei never display the ‘halfbeak’ morphology and predominantly lack elongate jaws (with the exception of some members of the genus *Adrianichthys*, which display jaw elongation that is either isometric (*A. poptae*) or even longer in the upper jaw (*A. krutyi* and *A. roseni*)) [31]. The flexibility of their ratio in jaw length appears to have promoted the evolutionary success of the Belonoidei compared to the Adrianichthyoidei, which include 240 and 33 species respectively, based on current estimates from FishBase (http://www.fishbase.org). This suggests an enhanced degree of evolvability, defined by Hansen as ‘the ability of the genetic system to produce and maintain potentially adaptive genetic variants’ [32]. Pronounced variation of jaw ratios in the Belonoidei, driven by growth heterochronies, appears to have promoted the exploitation of novel ecological niches over brief evolutionary timescales, which might explain their considerably greater species richness in comparison to their sister group.

While previous research has provided strong evidence that heterochrony has played a significant role in the evolution of beloniform fishes, it has not addressed the developmental or genetic bases of it. Importantly, previous studies on beloniform fishes did not compare their jaw development to that of medaka, a sister group [33] that has retained the ancestral condition of short upper and lower jaws, the character state that is observed in most fishes. Here we present a molecular developmental study of jaw elongation in the Beloniformes, with a comparative analysis of candidate gene expression during jaw outgrowth in halfbeak and medaka. The candidate genes include two markers of cartilage and bone development, *sox9* and *runx2* respectively, and genes associated with differential cellular proliferation in skeletal primordia, including *bmp4* and *calmodulin*, and *bmp2*. This approach enables a more informed interpretation of the significance of any changes in gene expression within a developmental framework.

## Methods

### Collection of juveniles

Adults of *Dermogenys pusilla*, a live-bearing species, were obtained through the aquarium trade in 2008, raised at a low density in well-planted tanks and fed flake food and frozen *Drosophila melanogaster*. Pregnant females were isolated in 40-litre tanks to give birth, to avoid cannibalization of the clutch by conspecifics. Larvae were collected at the required ages and euthanized with MS222, according to animal ethics requirements (Regierungspräsidiums Freiburg, Germany). Specimens used for skeletal analysis were fixed with 4% paraformaldehyde in 0.1 M PBS at 4°C, and stepped into 100% MeOH. Larval qRT-PCR samples were collected in the same way and stored in RNAlater (Qiagen, Stockach, Germany) until further processing. For the pre-birth stage, pregnant females were euthanized in MS222 and embryos were dissected from the uterus and stored in RNA later. Embryos of *Oryzias latipes* were collected using methods described in [34] and raised at 25°C with a few drops of methylene blue. Samples were fixed using the same methods as described for *D. pusilla*.

### Histochemistry and measurements

Skeletal staining was performed on fixed specimens using a modified alcian blue and alizarin red method, which uses acid-free alcian blue containing 60 mM MgCl_2_[35]. Three specimens for each time point were photographed with a Zeiss Stemi SV 11 microscope (Zeiss, Münich, Germany). Brightness and contrast were adjusted with Photoshop CS4 (Adobe Creative Cloud) and measurements of key features were made using ImageJ. Measurements of upper and lower jaw lengths were taken from the laterally oriented specimens, calculating the distance from the point of attachment between the maxilla and dentary, to the anterior tips of the premaxilla and dentary (toothless extensions in the case of the halfbeak and needlefish). We then analysed both heterochrony and allometry, using methods modified from Alberch *et al*. [9] and Boughton *et al*. [23]. The shape variable selected for our heterochrony analysis was the ratio between lower and upper jaws, as jaw ratio is considered to be an important shape measure, as this measure appears to relate to trophic niche according to previous studies [23,27,30]. We scaled the ages of all three species, similar to Alberch *et al.*[9] and Gould [36] such that onset age was birth or hatching for halfbeak and needlefish respectively and medaka were age-matched on the basis of skeletal ossification. Offset age was at completion of skeletal ossification for all three species. Allometry analyses (ontogenetic scaling) plotted growth of uj against lj. Additionally, 10 μM sections were cut for paraffin-embedded specimens and stained using acid-free alcian blue and alizarin red.

### Isolation of genes of interest

Candidate genes were isolated from cDNA of a single *D. pusilla* larva shortly after birth. Fragments of candidate genes were amplified using primers designed to alignments of teleost sequences obtained from GenBank (Additional file 2: Table S1) and cloned into either PGEM-T (Promega, Mannheim, Germany) or PCR-4 (Invitrogen, Darmstadt, Germany) vectors. RACE PCR and cloning was used to obtain 3′ UTR sequences. To determine the orthology of our candidate genes, we constructed maximum likelihood (ML) trees. Sequences were aligned to other vertebrate orthologues and paralogues in Jalview [37] using the MAFFT algorithm. After removal of gaps, ML trees were generated in PhyML [38], with 1,000 bootstrap replicates. *Calmodulin* orthologues were obtained from medaka through the GenBank database. To determine whether the genes of interest had undergone positive selection, we calculated the ratio between the number of non-synonymous substitutions (dN) per non-synonymous site versus the number of synonymous substitutions per synonymous site (dS) [39]. To achieve this we compared the halfbeak coding sequences to other teleost representatives, using yn00 in the PAML toolkit (v. 4.4) [40], selecting the Yang and Nielsen substitution model [41].

### qRT-PCR of candidate genes in the jaws of *Dermogenys*

The upper and lower jaws of specimens were excised under a dissecting microscope at the level of the nares (roughly corresponding to the point at which the maxilla is attached to the dentary). Upper and lower jaw samples include skeletal elements listed in Additional file 3: Table S2. RNA was extracted with the RNeasy Minikit (Qiagen, Stockach, Germany), using the optional on-column DNase treatment. Upper and lower jaws were dissected from multiple individuals and pooled for each stage, while biological replicates were kept separate. RNA quality was confirmed using agarose gel electrophoresis and purity and quantity were assessed with a NanoVue spectrophotometer (GE Healthcare, Freiburg, Germany). Prior to cDNA synthesis, we confirmed that gDNA contamination was negligible (when our samples were amplified alongside positive controls of comparable concentration, products were detected > ten cycles after the positive control). cDNA was synthesized from up to 200 ng of RNA with Superscript III (Invitrogen, Darmstadt, Germany). Primers were designed to our candidate genes using AmplifX 1.5.4 [42], with the reverse primer always in the 3′ UTR, maximizing the chance that they would amplify the intended transcripts, rather than orthologues or paralogues (Additional file 2: Table S1). Primer concentrations were individually optimized to minimize dimers (for all our primer combinations either no, or negligible dimer levels were detected) and standard curves were generated.

We performed qRT-PCR reactions with a BioRad C1000 Thermal Cycler, using iQ SYBR green Supermix (Bio-Rad, Münich, Germany) according to the manufacturer’s instructions. Average CT (avgCT) values were calculated across three technical replicates and corrected for PCR-efficiency (E = PCR-efficiency; E = 10^–(1/slope)^; relative expression = 1/E^avgCT^) of the corresponding primer pair as described in [43] and [44]. These raw values of relative expression of single individuals were then normalized to total RNA input determined with RiboGreen RNA assays (Invitrogen, Darmstadt, Germany) according to the manufacturers’ instructions. After confirming that the data had a normal distribution using the Shapiro-Wilks test, we used two-way ANOVA to determine whether jaw tissue or developmental stage, or an interaction between the two, influenced relative expression of our candidate genes.

### qRT-PCR of candidate genes in the jaws of medaka

RNA extraction was performed as described for halfbeak. In spite of using approximately ten specimens per tissue per timepoint, the yield was too low for reliable qRT-PCR (cDNA < 1 ng/reaction), thus our samples were amplified using the QuantiTect Whole Transcriptome Kit (Qiagen, Stockach, Germany). Initial trials in which two samples were amplified using *calm2* from the same template (n = 4) indicated that relative quantitation (RQ) could not be reliably scaled against RNA input quantity (average SD = 0.305). Instead, we developed a set of housekeeping genes (HKG), testing previous HKGs used for qRT-PCR on cichlid pharyngeal jaws [45], plus *β-actin* and *gapdh*. After optimisation with q-base, we selected three HKGs, including *Ubiquitin fusion degradation 1-like*, *β-actin* and *gapdh*. Normalising RQ against these HKGs (RQ candidate/mean RQ HKGs) resulted in a significant reduction in the SD of samples run from the same templates (average. SD = 0.141). All templates were then normalised to these three HKGs, for each primer combination (Additional file 2: Table S1). We analysed the expression of all genes included in the halfbeak analysis with the exception of *runx2*, which could not be reliably amplified in medaka.

## Results

### Characterizing growth heterochronies in halfbeak and needlefish

The relationship between jaw shape and age was examined in *D. pusilla*, *O. latipes* and *B. belone*, through plotting ratios between lower and upper jaws against scaled age using a method modified from [23]. At onset age, all three species have equally long upper and lower jaws (that is, jaw ratios of close to 1.0) (Figure 1B). The upper and lower jaws of medaka continue to display isometric growth, representing the ancestral condition (Figure 1B, C). Halfbeak and needlefish both undergo accelerated lower jaw growth, as indicated by lower jaw to upper jaw ratios (lj:uj) that peak at approximately 1.8 and 3.0 respectively (Figure 1B, C). In needlefish, on the other hand, jaw development displays a second phase in which uj growth accelerates in relation to lj until they attain equal length, a proportion that is then maintained throughout their lives (Figure 1B, C). Halfbeak lj:uj is then maintained at approximately 1.8 through to adulthood (data not shown). As needlefish jaw growth includes two age-dependent slopes prior to offset age (which is also when halfbeak jaws reach their adult ratio), we consider needlefish jaw development to represent an example of mosaic heterochrony [46]. Ontogenetic scaling analysis comparing uj and lj length demonstrates that the medaka lj grows isometrically with the uj, while halfbeak and needlefish display peramorphosis in lj growth, due to a positive shift in the slope of their trajectories in comparison to the ancestral medaka slope (Figure 1D, E). Additionally, we demonstrate that needlefish jaw growth displays an inflection later in development, displaying a negative slope that brings the uj and lj back to an isometric ratio. This is due to a secondary acceleration of uj growth, rather than a reduction in the rate of lj growth, as indicated by allometric growth analyses that plot uj and lj length against log body length (Additional file 4: Figure S2).

### Examination of skeletal development

**Figure 2:**
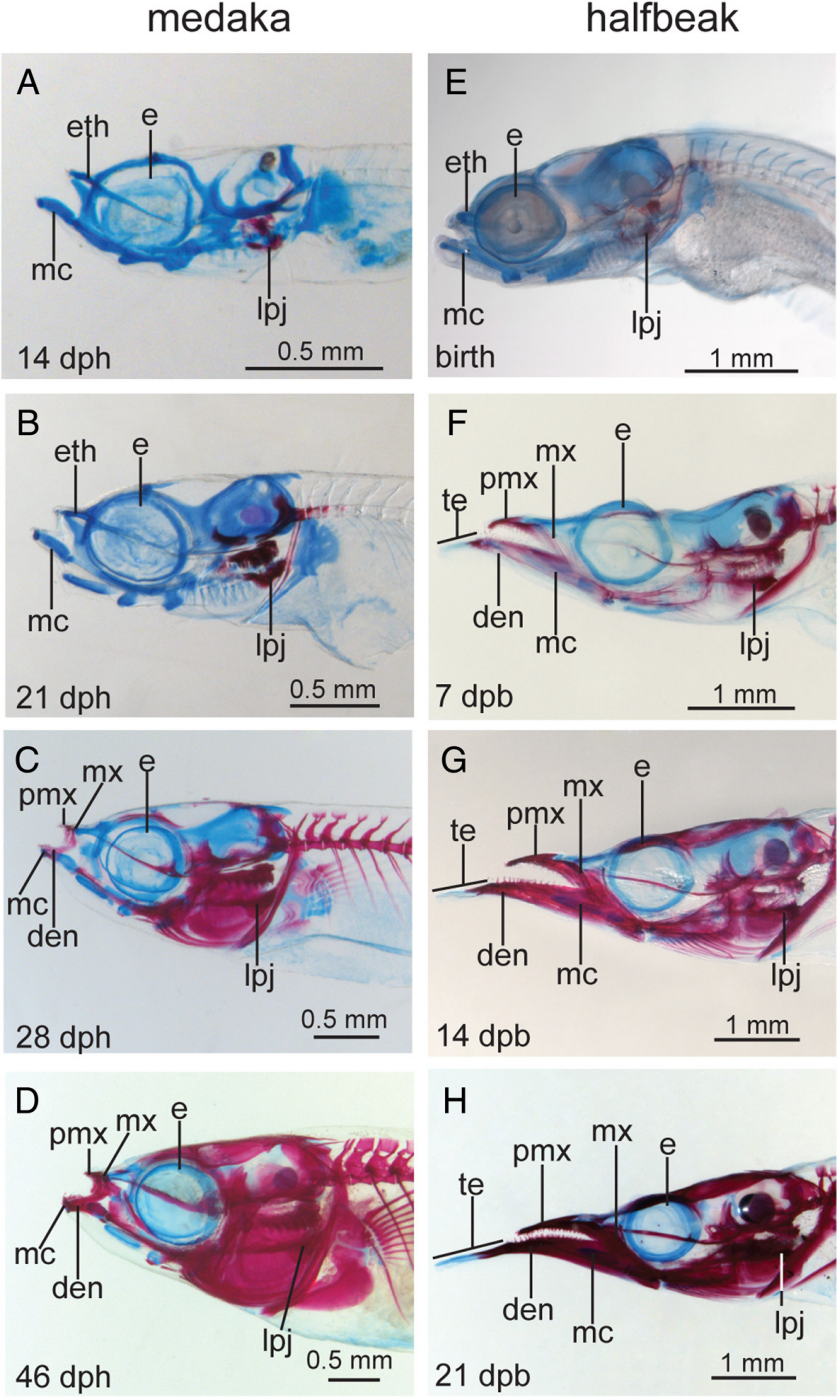
**Comparative skeletal development of medaka, *Oryzias latipes *and halfbeak, *Dermogenys pusilla. *(A-H)** lateral orientation, **(A-D) ***O. latipes*, **(E-H) ***D. pusilla*, stages are matched based on the relative timing of skeletal ossification. The upper and lower jaws of medaka maintain roughly similar proportions throughout development **(A-D)**. At birth, the jaws of halfbeak resemble those of medaka **(E)**, however they become elongated throughout development, whereby the lower jaw grows proportionally more than the upper jaw **(F-H)**. Abbreviations: den = dentary, dpb = days post birth, dph = days post hatch, e = eye, eth = ethmoid, lpj = lower pharyngeal jaw, mc = Meckel’s cartilage, mx = maxilla, pmx = premaxilla.

The developmental basis of lower jaw extension was examined using histostaining, comparing needlefish and halfbeak development to that of medaka, with a particular focus on halfbeak and medaka due to their experimental tractability. As skeletal development has previously been described for these three species [47-49], aspects of skeletal development that related directly to growth heterochrony were the main focus of this study. Halfbeak and needlefish at birth and hatching respectively, displayed a similar jaw morphology to age matched medaka (Figure 2A and E, data not shown). One notable exception is that compared to medaka, the Meckel’s cartilages of halfbeak and needlefish display a shift in their posterior limits, corresponding to a posterior fusion of the angulo-articular to the dentaries in halfbeak and needlefish [47] (conversely, the angulo-articular is nested under the dentaries in medaka).

**Figure 3:**
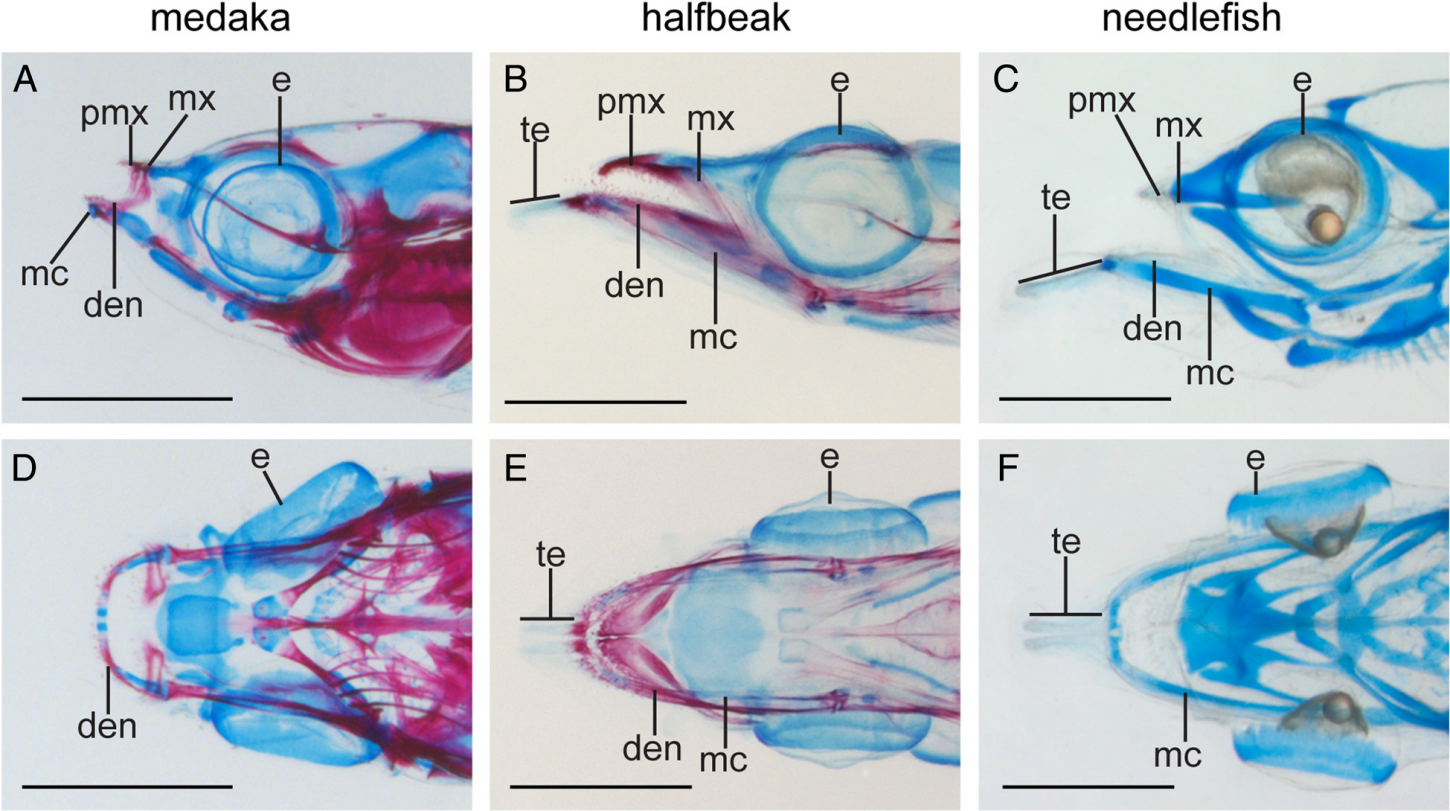
**Contribution of the toothless extensions to heterochronic jaw growth in representatives of the Belonoidei.** Alcian blue/alizarin red staining indicates that needlefish (*B. belone*) and halfbeak (*D. pusilla*) have toothless extensions of the lower jaw that are not present in medaka (*O. latipes*). **(A, C, E)**, lateral orientation, **(B, D, F)** ventral orientation; age matched representatives, including **(A, B)** 28 dph medaka, *O. latipes*, **(C, D)** 7 dpb halfbeak, *D. pusilla*, **(E, F)** 6 dph needlefish, *B. belone*. Abbreviations: bl = body length, e = eye, lj = lower jaw, ljb = lower jaw basis, te = toothless extensions, uj = upper jaw.

Shortly after birth/hatching, halfbeak and needlefish display two rostrally-oriented outgrowths of the dentaries at their rostro-median contact zones (Figure 2 F, Figure 3C-F, Additional file 5: Figure S3). The dentary outgrowths continue to extend throughout development and do not bare any teeth, even in mature adult stages (Figure 2 F-H, data not shown). Hence, they will hereon be referred to as ‘toothless extensions’. At early developmental stages, the toothless extensions do not stain with alizarin red, and only faintly with alcian blue, which is not visible in stained histological sections (Additional file 5: Figure S3). The toothless extensions become progressively more ossified throughout development in a proximo-distal direction, (Figure 2 F-H, Additional file 5: Figure S3), however the distal tips remain unossified even in adults (Figure 2G-H, data not shown) [47,50].

**Figure 4:**
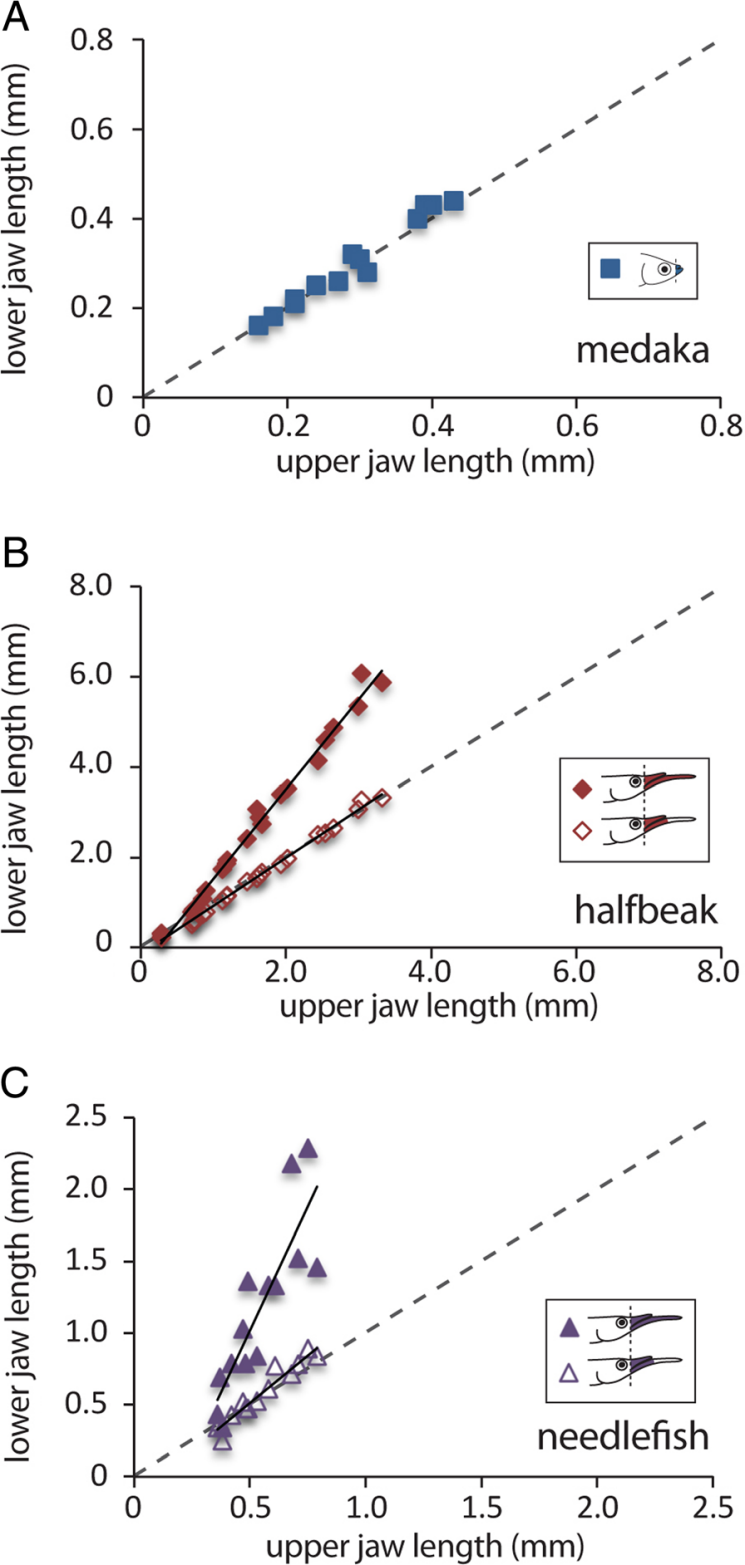
**The toothless extensions form the basis for heterochronic shifts in jaw growth amongst the Belonoidei.** Allometric plots displaying ratios between upper and lower jaw growth for **(A)** medaka, **(B)** halfbeak, and **(C)** needlefish as well as ratios between upper and lower jaw excluding the toothless extensions for **(B)** halfbeak and **(C)** needlefish. **(A)** The jaws of medaka, which display the ancestral condition, undergo isometric growth (represented by the dashed line). **(B)** Comparison between upper and lower jaw growth for halfbeak indicates growth acceleration in favour of the lower jaw, while comparison between upper and lower jaw excluding the toothless extensions indicates isometric growth (represented by the dashed line). **(C)** A similar pattern to halfbeak is observed in needlefish. Note, needlefish growth trajectory is based on only the data points prior to growth acceleration of the upper jaw.

The toothless extensions appear to be morphological innovations that are critical to establishing growth heterochrony amongst the Belonoidei. No such structure is observed in medaka, while they are present in both halfbeak and needlefish, and are therefore likely to be present throughout the Belonoidei (Figure 3A-F). While the teeth on the halfbeak dentary articulate with those of the premaxilla (meaning that there is an otherwise isometric relationship between uj and lj), it is the toothless extensions that project the lower jaw beyond the anterior limit of the premaxilla (Figure 4A-C).

### qRT-PCR profiling of candidate gene expression during jaw outgrowth

**Figure 5:**
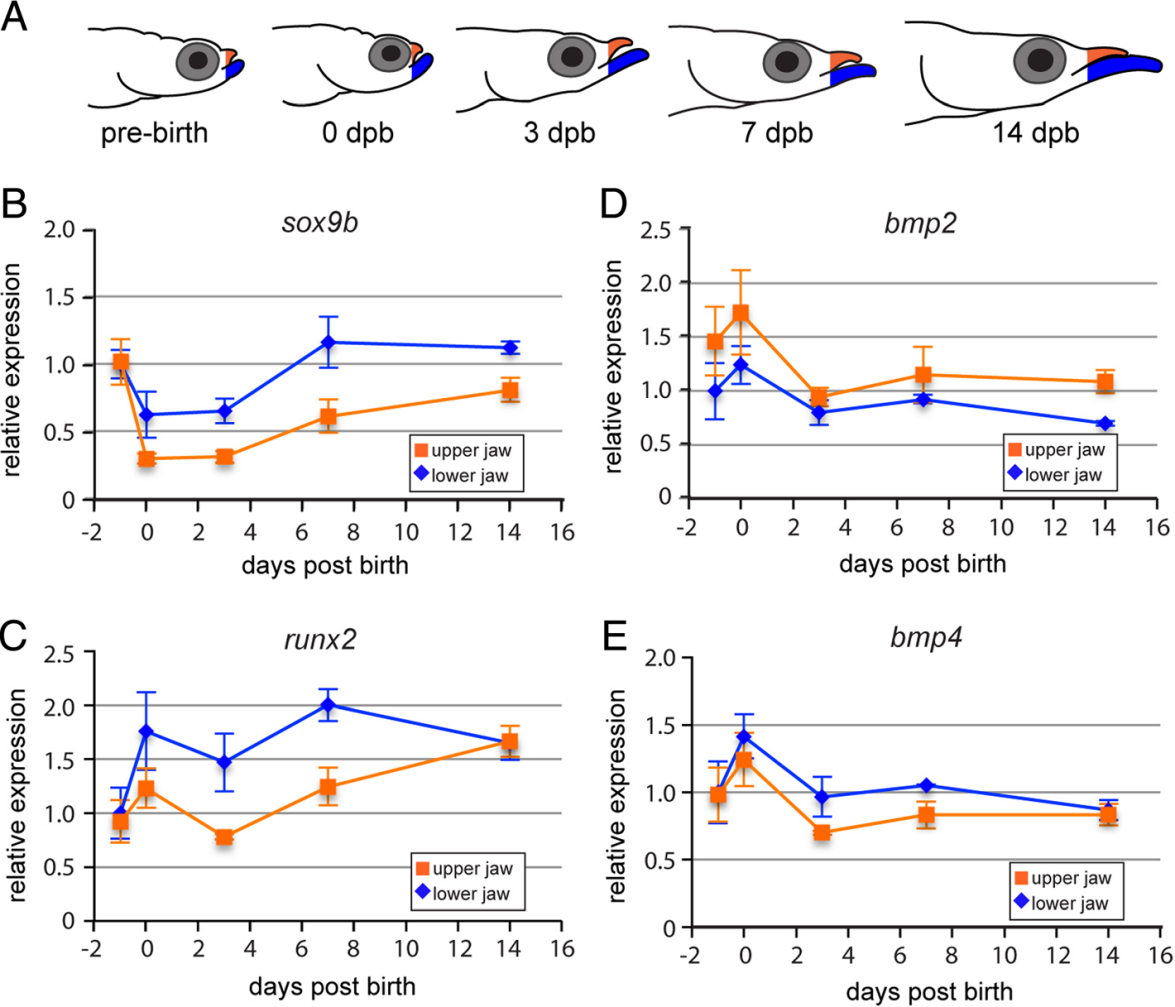
**Expression of candidate genes during jaw outgrowth in *Dermogenys pusilla. *(A)** Cartoon of tissue sampling for the expression analyses. Upper and lower jaws were excised at the level indicated by the orange and blue colouring respectively, from stages indicated. **(B-E)** Expression of candidate genes **(B) ***sox9b*, **(C) ***runx2*, **(D) ***bmp2* and **(E) ***bmp4*. Relative expression values are scaled so that expression in the lower jaw in embryos = 1. Standard error is indicated. Two-way ANOVAs indicated statistically significant differences in expression for *sox9b* (*P* < 0.001), *runx2* (*P* < 0.01) and *bmp2* (*P* < 0.05) but not *bmp4* (*P* > 0.1) (see Table 1). RQ = relative quantitation.

**Figure 6:**
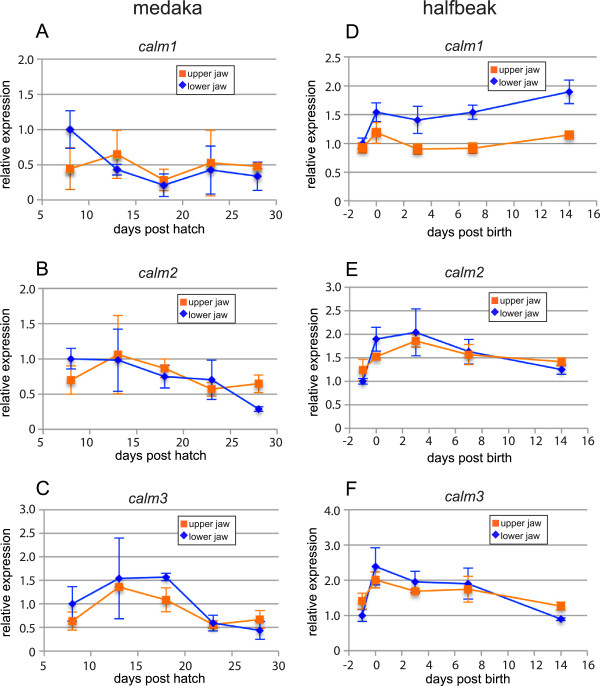
**Expression of *calm *paralogues during jaw outgrowth in medaka and halfbeak. (A-F)** Expression of *calm* paralogues in **(A-C)** medaka and **(D-F)** halfbeak, including **(A, D) ***calm1*, **(B, E) ***calm2*, **(C, F) ***calm3*. Note, genes we denote medaka *calm1*, *calm2* and *calm3* have previously been denoted *CaM-A*, *CaM-B* and *CaM-C*, but we have re-named them due to the orthology relationships identified by our phylogenetic analysis. Relative expression values are scaled so that expression in the lower jaw of embryos = 1. Standard error is indicated. Two-way ANOVAs show a statistically significant difference in expression for *calm1* in halfbeak (*P* < 0.001), but not medaka (*P* > 0.1). Neither *calm2* nor *calm3* show differential expression for halfbeak or medaka (*P* > 0.1) (see Table 1). RQ = relative quantitation.

The molecular basis of heterochronic growth was examined in halfbeak as a first step to determining the molecular basis of craniofacial diversity within the Belonoidei. The expression of candidate genes, including *bmp2*, *bmp4, runx2*, *sox9b* and three *calmodulin* orthologues, denoted *calm1, calm2* and *calm3* was compared between upper and lower jaws at a range of developmental stages that appear to be important for jaw outgrowth (Figure 5A, Additional file 6: Figure S4, Additional file 3: Table S2). We observed significantly differential expression for four of the seven genes examined, based on two-way ANOVAs (Table 1). The majority of genes in our study were upregulated in the lower jaw relative to the upper jaw - amongst these were *sox9b*, *runx2* and *calm1*, while *bmp2* was upregulated in the upper jaw (Figure 5B-D, Figure 6D). As both *sox9b* and *runx2* were upregulated in the lower jaw, we infer that both chondrogenic (cartilage-forming) and osteogenic (bone-forming) pathways are required for the outgrowth of the lower jaw, presumably the result of their enhanced expression in Meckel’s cartilages and the dentaries respectively (Additional file 3: Table S2). Expression of *bmp2* was significantly higher in the upper jaw, which matches neither *runx2* nor *sox9b* (Figure 5B-D), reducing the possibility that it is involved in either bone or cartilage development for the stages we examined.

**Table 1:** Results of two-way ANOVA on qRT-PCR of skeletogenic genes

**Organism**	**Gene**	**Jaw (*P*-value)**	**Age (*P*-value)**	**Interaction (*P*-value)**
Halfbeak	*runx2*	**0.005**	**0.009**	0.248
Halfbeak	*sox9b*	**0.001**	**> 0.001**	0.255
Halfbeak	*bmp2*	**0.020**	**0.048**	0.909
Halfbeak	*bmp4*	0.132	**0.016**	0.869
Halfbeak	*calm1*	**> 0.001**	**0.011**	0.209
Halfbeak	*calm2*	0.780	**0.014**	0.666
Halfbeak	*calm3*	0.976	**0.005**	0.528
Medaka	*sox9b*	0.167	0.118	0.284
Medaka	*bmp2*	0.282	0.192	0.302
Medaka	*bmp4*	0.283	**0.045**	0.370
Medaka	*calm1*	0.243	0.099	0.238
Medaka	*calm2*	0.257	**0.016**	0.206
Medaka	*calm3*	0.280	0.080	0.363

Significant *P*-values are highlighted in bold (*P* < 0.05).

Interestingly, the three *calm* genes displayed differential patterns of expression, where *calm1* was the only *calm* gene whose expression differed significantly between the upper and lower jaws based on two-way ANOVAs (Figure 6D-F, Table 1). For *calm2* and *calm3*, expression remained equal between the upper and lower jaws throughout development (Figure 6E and F). Unlike all other genes investigated, the relative expression of *calm1* progressively increases in the lower jaw as development progresses in a pattern that matches jaw outgrowth (see accelerated jaw growth for halfbeak in Figure 1B; Additional file 7: Figure S5). Moreover, medaka does not show significant differences in the expression of *calm1*, *calm2* or *calm3* in the jaws during development (Figure 6A-C, Table 1), providing further evidence that *calm1* may control jaw growth in halfbeak. Additionally, none of the investigated genes displayed differential expression between upper and lower jaws in medaka. We consider *calm1* to be a promising candidate for heterochronic growth of the lower jaw in halfbeak and by extension, of craniofacial diversification in the Belonoidei. All genes included in this analysis were under purifying selection (dN/dS ratios < 1), indicating that any regulatory evolution displayed by our genes of interest is not accompanied by positive selection in their coding regions.

## Discussion

We characterized the molecular basis of heterochronic shifts that underlie striking craniofacial novelties in some lineages of beloniform fishes. Previous studies of heterochrony in this group merely dealt with the overall jaw length without taking into account the skeletal elements that contributed to jaw length, or the differential expression of genes that control their growth [23,27,51]. Our results demonstrate that different rates and directions of acceleration in jaw growth have shaped the evolutionary transitions from medaka to halfbeak, and halfbeak to needlefish morphologies respectively. Through normalizing developmental stage based on skeletal ossification, our heterochrony investigations arrive at a different conclusion than previous studies [2], classifying needlefish jaw development as undergoing two phases of acceleration (mosaic heterochrony) rather than hypermorphosis. Moreover, we identify toothless extensions of the dentaries as an important morphological innovation that shapes jaw heterochrony within the Belonoidei. *calm1* is identified as a potential molecular determinant of this craniofacial innovation in the Belonoidei. Interestingly, *calm* has been shown to play a role in establishing jaw (and beak) morphology in well-known adaptive radiations such as Galapagos finches and cichlid fish [17,22]. Our results provide evidence that *calm* may be an evolutionarily widely utilized regulator of craniofacial diversity in the vertebrates.

The toothless extensions of the dentaries of the Belonoidei represent an important morphological innovation, which is likely to have shaped the craniofacial diversity within this group, expanding their evolutionary potential and their ‘evolvability’ [52]. Forming a terminal addition to the dentaries, the toothless extensions represent a discrete and flexible module that displays ‘weak linkage’, one of the key features of evolvable systems identified by Kirschner and Gerhardt [52]. Moreover, the toothless extensions are likely to represent an important innovation amongst the Belonoidei due to their presence in both halfbeak and needlefish. This flexibility in jaw length appears to have enabled the Belonoidei to exploit new ecological niches over rapid evolutionary timescales. Indeed, the suborder Belonoidei includes almost an order of magnitude more species than Adrianichthyoidei (240 and 33 species respectively), which lack this evolutionary innovation.

The importance of the toothless extensions as an evolutionary innovation lies in the highly flexible nature of their development and of their ultimate length in the adult, attested by the diversity of jaw phenotypes in the suborder Belonoidei. For example, there are even remarkable differences in lower jaw length among closely related halfbeak species [53,54]. In some halfbeak and flying fish genera the lower jaw extension is secondarily resorbed after its initial outgrowth in the larval stage [53] and lower jaw outgrowth has been entirely lost in most flying fish genera, as inferred from a phylogenetic reconstruction of their ancestral states [27,51]. It seems plausible that developmental pathways controlling cellular proliferation, differentiation and apoptosis in the toothless extensions contribute directly to diversity of jaw phenotype in this group.

In an effort to better understand the molecular determinants of jaw elongation in halfbeak, expression of a range of skeletogenic genes was analysed in several developmental stages during jaw outgrowth. We observed dynamic patterns of gene expression for many of these, the majority of which indicated upregulation in the lower jaw relative to the upper jaw - amongst these were *sox9b*, *runx2* and *calm1* - while *bmp2* was upregulated in the upper jaw (Table 1). As both *sox9b* and *runx2* were upregulated in the lower jaw, we infer that both chondrogenic (cartilage-forming) and osteogenic (bone-forming) pathways are required for the outgrowth of the lower jaw. This pattern is likely to be the result of their enhanced expression in Meckel’s cartilages and the dentaries respectively, potentially including the toothless extensions. Future studies of spatial expression patterns are required to determine if this is the case. Elevated relative expression of *bmp2* in the upper jaw, when both osteogenesis and chondrogenesis are enhanced in the lower jaw, suggests that *bmp2* does not contribute significantly to lower jaw outgrowth in halfbeak. Alternatively, expression of *bmp2* may be strong in the teeth (which are at various stages of development at birth) rather than the jaws, as *bmp2* has a conserved role in teleost dentition [55]. The upper jaw samples are composed of a relatively higher proportion of tooth germs, as the lower jaw also includes toothless extensions, potentially diluting the *bmp2* mRNA concentration over the entire sample.

We consider *calm1* to be a strong candidate for elevated outgrowth of the lower jaw of *D. pusilla*, and by extension, of craniofacial diversification in the Belonoidei. Expression of *calm1* showed a unique pattern compared to its two paralogues, *calm2* and *calm3*, suggesting that *calm1* may have undergone regulatory evolution in the Belonoidei, resulting in outgrowth of the lower jaw. Moreover medaka, which has an ancestral jaw morphology, displays equal expression of *calm1*, *calm2* and *calm3* in the upper and lower jaws, strengthening the association between elevated relative *calm1* expression and lower jaw outgrowth. Previous studies suggested that calm promotes craniofacial novelties in cichlid and finch through different developmental mechanisms, which may shed light on its role in halfbeak. For cichlids, *calm* expression was increased in the dentaries of deep-jawed species, with a proposed role in increasing osteoblast proliferation [22], while in finches with robust beaks, *calm* expression was increased in the distal mesenchyme of the frontonasal prominence, which appeared to increase the length of the pre-nasal cartilage rod [17]. As the dentary develops through intramembranous rather than endochondral ossification, we predict that similar to cichlid, overexpression of *calm1* in the lower jaw of the halfbeak directly increases osteoblast proliferation, promoting jaw elongation, however *in situ* hybridization is required to determine whether this is the case. Osteoblast proliferation may be promoted by calm, through increased activation of Calcium/calmodulin-dependent kinase II (CdKII) [56], which controls the expression of osteoblast proliferation and differentiation-related genes such as *Osterix*[57] and *dlx5*[58]. To date, it is unclear whether a single *calm* orthologue underlies morphological novelties amongst halfbeaks, finches and cichlids, as our phylogenetic analyses could not assign clear orthology to the finch *calm* paralogue (data not shown), and the cichlid *calm* sequence has not been made publically available.

In line with other vertebrates, the halfbeak and medaka genomes include multiple *calm* genes that encode proteins with identical amino acid sequences (for a review see [59]; see also [60-64]). The retention of such apparently redundant paralogues in the genome may serve a functional purpose, allowing for *calm* expression to be fine-tuned in a spatially and temporally appropriate manner, maintaining tight regulation of calcium sensitive pathways. For example, studies of *calm* expression in the rat brain demonstrate that they differ spatially [65,66], temporally [66,67] and in response to cortical lesions [68] and reserpine, an inhibitor of catecholaminergic neurotransmitters [69]. Our results suggest that *calm1* has undergone regulatory evolution after the split between Adrianichthyoidei and Belonoidei, leading to its increased expression in the lower jaw. We hypothesize that the increased expression of *calm1* locally increases the activation of calcium sensitive pathways, activating osteoblast proliferation, which leads to lower jaw elongation [70-72].

Our results suggest that *calm1* expression controls the heterochronic growth of a highly modular and developmentally flexible trait, the toothless extension, potentially promoting enhanced trophic differentiation amongst the Belonoidei. The variable growth rates of the jaws of Belonoid fishes allows us to test this hypothesis - for example, as the ratio between the lower and upper jaws is higher in *B. belone* than in *D. pusilla*, we predict that the ratio of relative expression between lower and upper jaws would be higher in *B. belone* than *D. pusilla*. It would be particularly useful to test this hypothesis in beloniform genera that display marked differences in the relative lengths of their upper and lower jaws, such as *Nomorhamphus* spp. [54].

In their landmark paper on evolvability, Kirschner and Gerhardt highlighted the Calmodulin pathway as a particularly evolvable system due to the flexibility in its binding to target proteins [52]. Our study demonstrates a further feature of Calmodulin that may promote evolvability: a reduced level of pleiotropy of this essential pathway, achieved through the retention of duplicated gene copies that encode identical proteins. This may explain why Calmodulin has been repeatedly utilised in the development of diverse craniofacial innovations [17,19,22]. This allows for regulatory evolution to act on individual gene copies, while the functions of the other copies remain unaffected. The presence of multiple *calm* gene copies in the genome extends the modularity of *calm* expression through reducing the pleiotropy of this pathway. This feature of Calmodulin may help to explain its repeated implication in the generation of morphological novelties amongst vertebrates [17,22].

## Conclusions

Heterochronic shifts in growth can result in dramatic alterations to morphology, which sometimes lead to a new adaptive peak via a simple developmental switch. Our fine-scale study of growth heterochrony in three beloniform fish species identified toothless extensions of the dentaries as an important source of heterochronic growth amongst the Belonoidei. This suborder is considerably more speciose than its’ sister group the Adrianichthyoidei, which has retained the ancestral morphology. Regulatory evolution of *calm1* is likely to contribute to heterochronic growth of the lower jaw amongst the Belonoidei, facilitating their impressive diversification and promoting their evolutionary success.

## Abbreviations

bp: base pairs; CdKII: Calcium/calmodulin-dependent kinase II; CT: threshold cycle; dpb: days post birth; dph: days post hatch; eth: ethmoid; HKG: housekeeping gene; lj: lower jaw; mc: Meckel’s cartilage; ML: maximum likelihood; mx: maxilla; pmx: premaxilla; RQ: relative quantitation; te: toothless extensions; uj: upper jaw.

## Competing interests

The authors declare that they have no competing interests.

## Authors’ contributions

AM and HG designed the study. HG and CK conducted the laboratory work. HG wrote the first draft and all authors revised the manuscript, and approved it for final publication.

## Authors’ information

Helen Gunter is a postdoctoral fellow in the Meyer lab and in the Zukunftskolleg of the University of Konstanz, Germany. She investigates the environmental and genomic interactions that shape animal phenotypes, focusing primarily on teleost craniofacial development. Claudia Koppermann conducted her diploma research on halfbeak development at the University of Konstanz. Axel Meyer is an evolutionary biologist interested in the origin of diversity at different levels of biological organization.

## Supplementary Material

Additional file 1: Figure S1 Examples of heterochronic shifts in growth. Click here for file

Additional file 2: Table S1 Primers used in this study. Click here for file

Additional file 3: Table S2 Skeletal elements contributing to length measurements. Click here for file

Additional file 4: Figure S2 Further investigation of heterochrony and allometry. Click here for file

Additional file 5: Figure S3 Histological analysis of jaw development in *D. pusilla.* Click here for file

Additional file 6: Figure S4 Phylogenetic analysis of genes cloned from *D. pusilla.* Click here for file

Additional file 7: Figure S5 Ratio of gene expression in upper and lower jaws of *D. pusilla.* Click here for file
