# Supplementary figures and images for: Revisiting de Beer’s textbook example of heterochrony and jaw elongation in fish: calmodulin expression reflects heterochronic growth, and underlies morphological innovation in the jaws of belonoid fishes
Source: EvoDevo. 2014 Feb 5;5:8. doi: 10.1186/2041-9139-5-8 (PMC3927394; doi:10.1186/2041-9139-5-8)

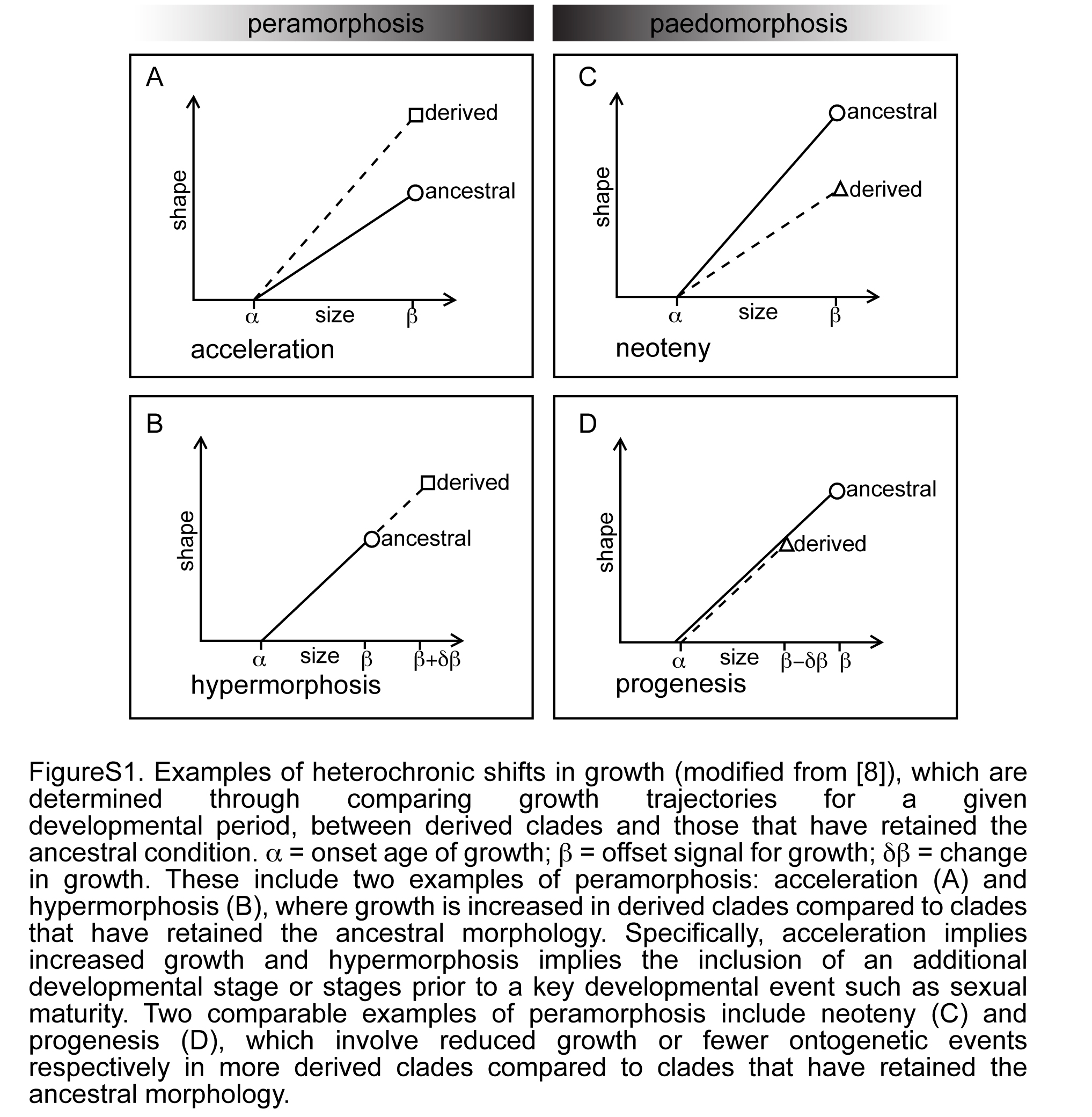

Supplement: Additional file 1: Figure S1 — Examples of heterochronic shifts in growth. [file 2041-9139-5-8-S1.jpeg]

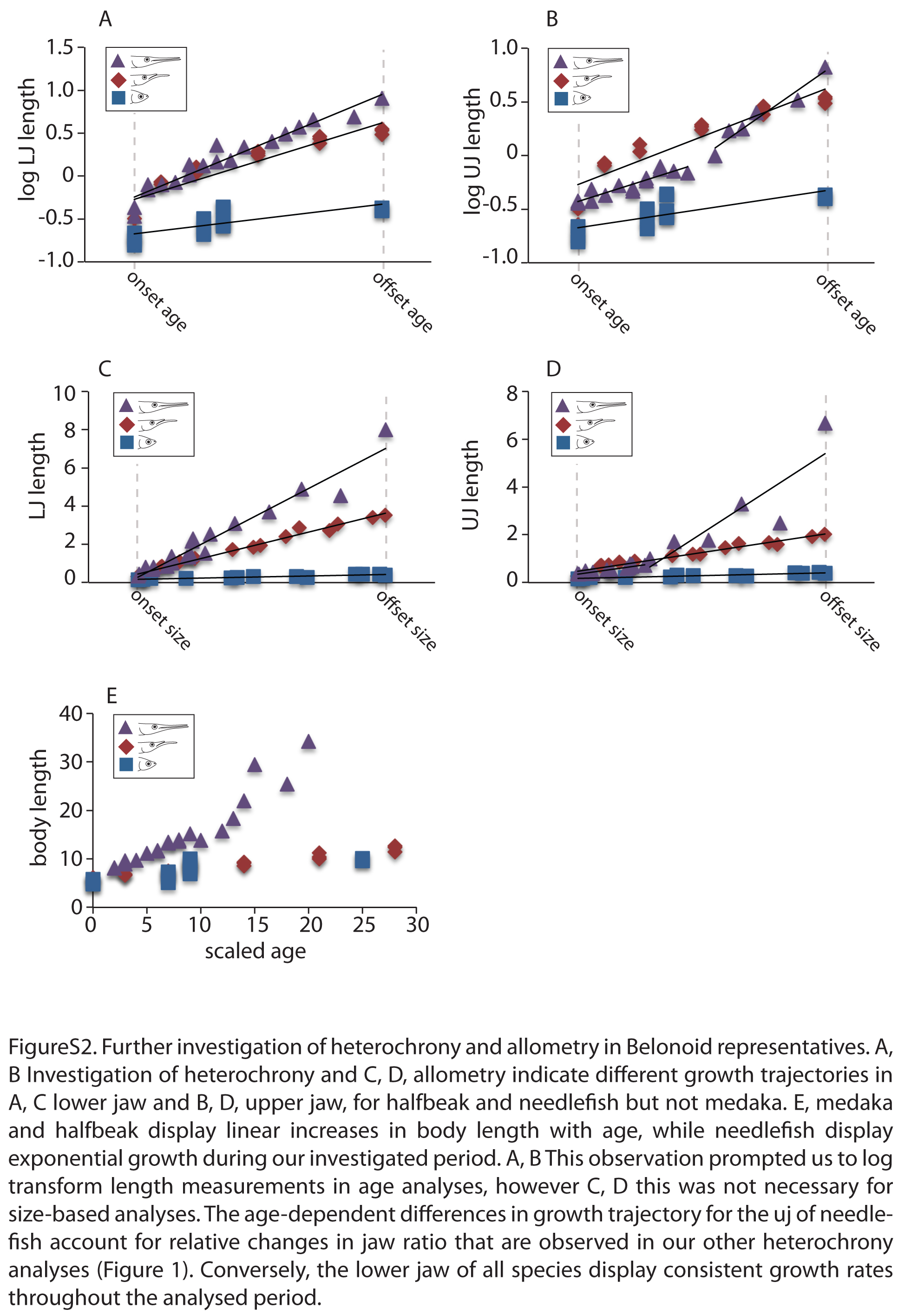

Supplement: Additional file 4: Figure S2 — Further investigation of heterochrony and allometry. [file 2041-9139-5-8-S4.jpeg]

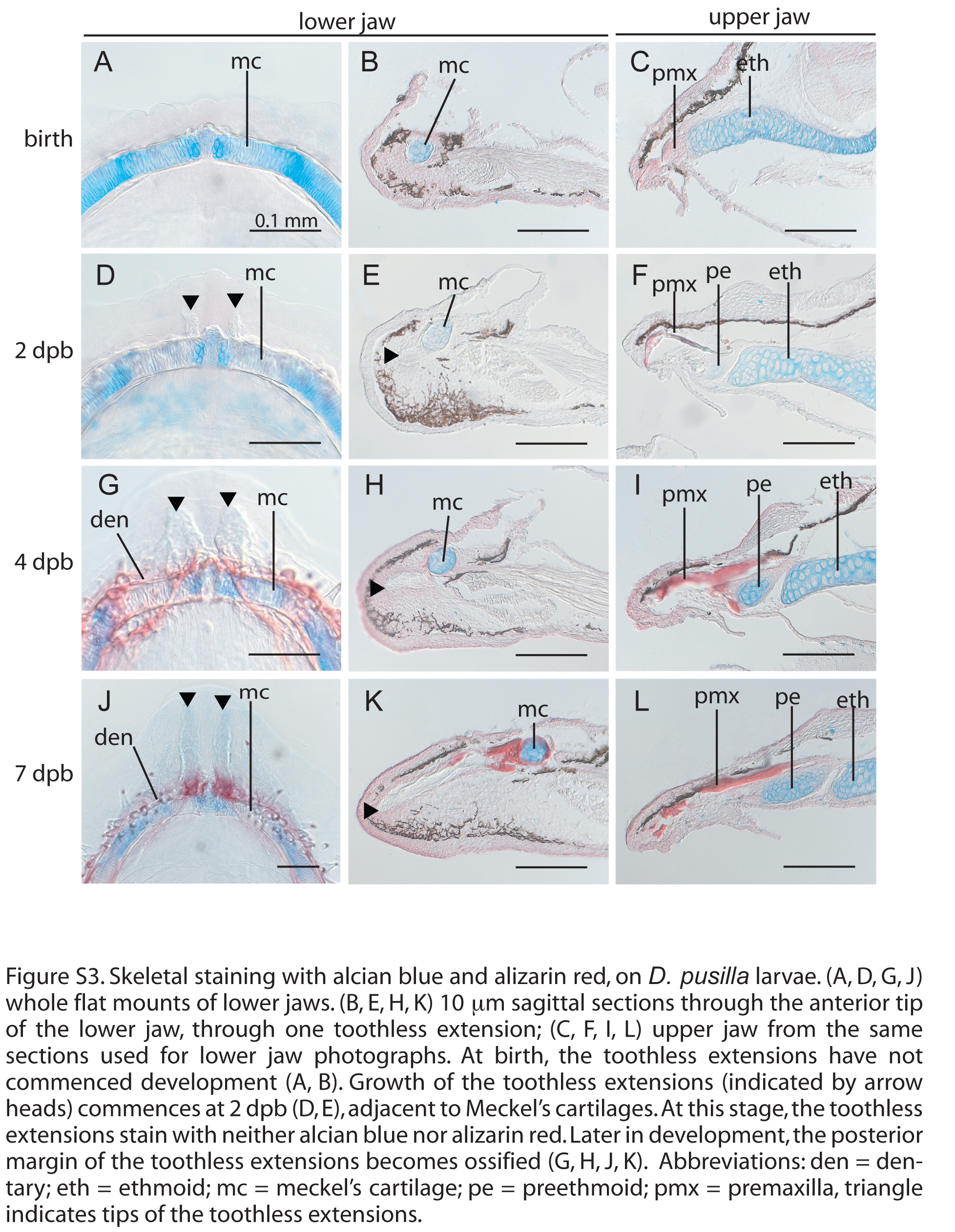

Supplement: Additional file 5: Figure S3 — Histological analysis of jaw development in D. pusilla. [file 2041-9139-5-8-S5.jpeg]

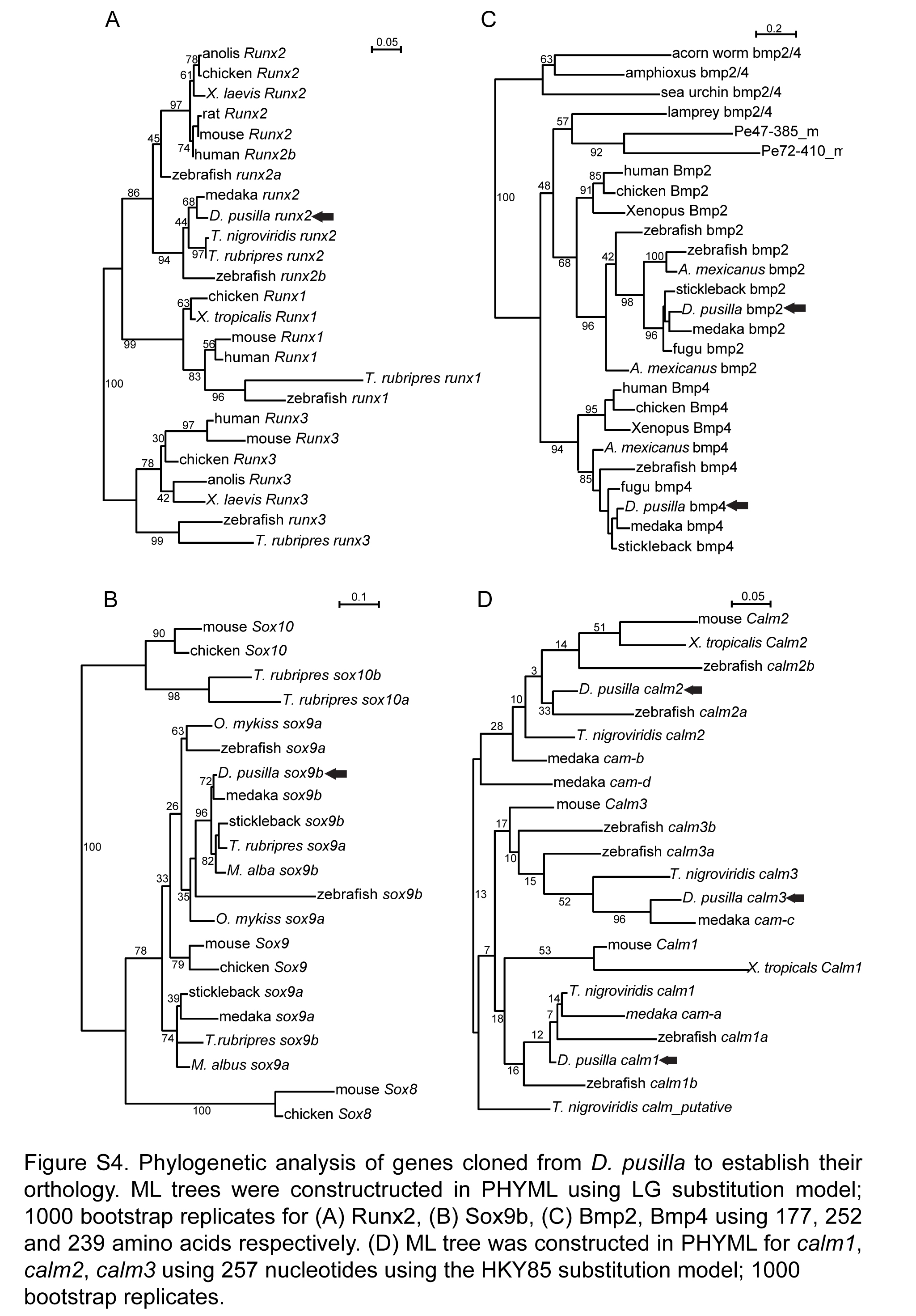

Supplement: Additional file 6: Figure S4 — Phylogenetic analysis of genes cloned from D. pusilla. [file 2041-9139-5-8-S6.jpeg]

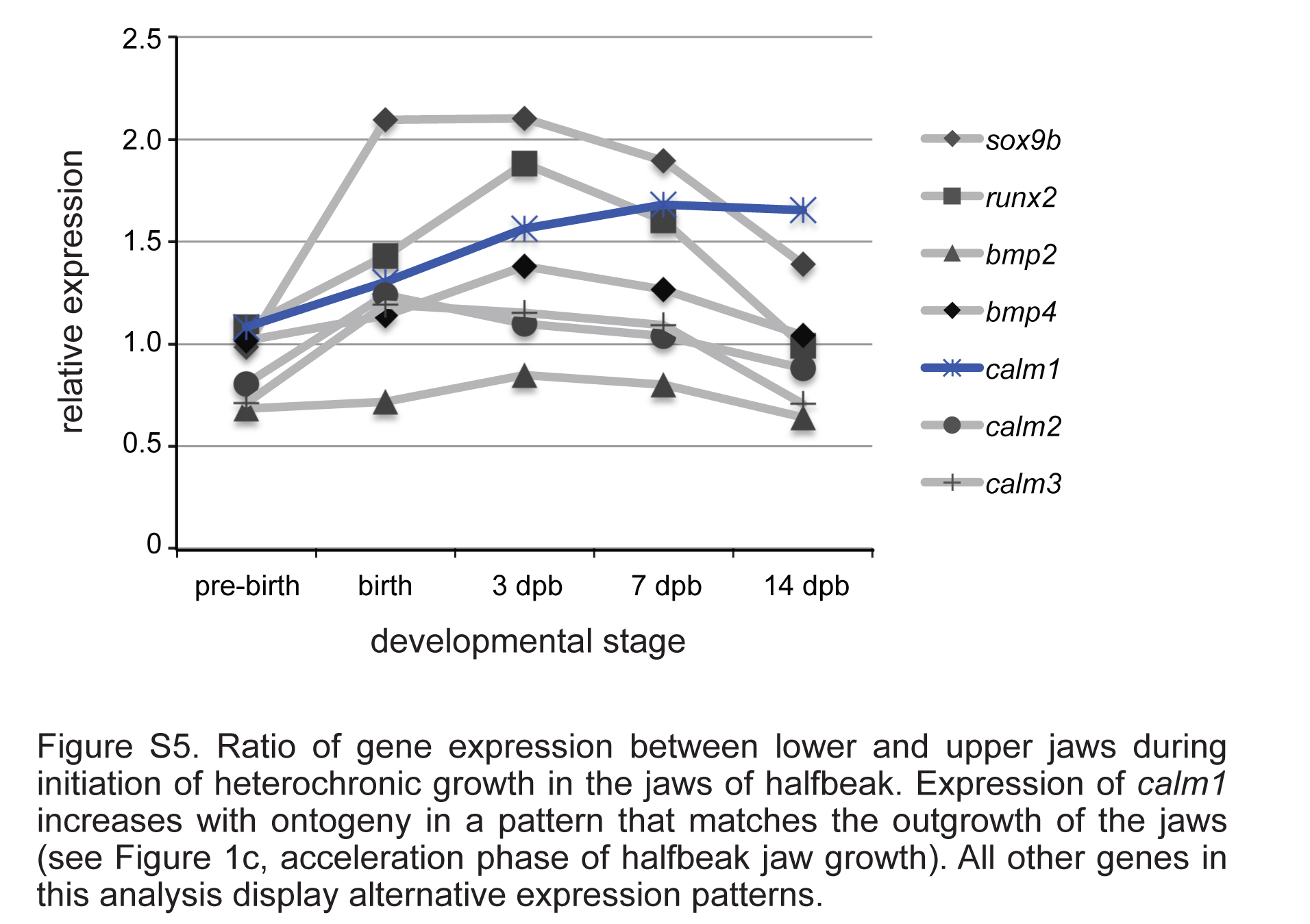

Supplement: Additional file 7: Figure S5 — Ratio of gene expression in upper and lower jaws of D. pusilla. [file 2041-9139-5-8-S7.jpeg]
